# Identification of a major facilitator superfamily protein that is beneficial to L-lactic acid production by *Bacillus coagulans* at low pH

**DOI:** 10.1186/s12866-022-02736-2

**Published:** 2022-12-20

**Authors:** Wenzhe Tian, Jiayang Qin, Congcong Lian, Qingshou Yao, Xiuwen Wang

**Affiliations:** College of Pharmacy, Binzhou Medical University, Yantai, 264003 China

**Keywords:** *Bacillus coagulans*, L-LA production, Genome sequencing, Major facilitator superfamily

## Abstract

**Background:**

Product inhibition is one of the major problems in lactic acid (LA) fermentation. Our previous study revealed that *Bacillus coagulans* 2–6 was an efficient producer of high-optical-purity L-LA. Its mutant strain *B. coagulans* Na-2 has better resistance to sodium lactate stress but the resistance mechanism has not been understood.

**Results:**

In this study, the whole-genome sequencing of *B. coagulans* Na-2 was performed and one mutant gene *mfs* coding for the major facilitator superfamily (MFS) protein was revealed by comparative genome analysis. Ten mutation sites were identified between the wild (MFS-2-6) and mutant (MFS-Na-2) proteins, among which T127A and N154T were predicted locating in the center of the transmembrane transport channel. The MFS-2-6 and MFS-Na-2 were expressed separately in a genetically operable strain, *B. coagulans* DSM1, using the genes’ native promoter. The expression of the two MFS proteins had no effect and a negative effect on L-LA production when the pH was controlled at 6.0 and 7.0 by sodium hydroxide, respectively. However, 4.2 and 4.6-fold of L-LA concentrations were obtained at pH 5.0 by the strains expressing MFS-2-6 and MFS-Na-2 than that by the control strain, respectively. The intracellular pH values of the strains expressing MFS-2-6 and MFS-Na-2 were approximately 0.69 and 0.45 higher than that of the control strain during pH-controlled fermentation at 5.0. Results suggest that the expression of MFS-2-6 and MFS-Na-2 were both conducive to L-LA production at low pH, while the better performance of the latter was probably due to the more appropriate intracellular pH during the whole fermentation process.

**Conclusions:**

The MFS protein identified here can improve the ability of *B. coagulans* to resist acidic environments and produce more L-LA at low pH. The MFS protein has an application potential in environment-friendly L-LA production.

**Supplementary Information:**

The online version contains supplementary material available at 10.1186/s12866-022-02736-2.

## Background

Lactic acid (LA), also known as dihydroxy propionic acid, is one of the world’s three major organic acids. LA is a small molecular platform compound and widely used in food, chemical, pharmaceutical, and other industries [1, 2]. LA has received increasing attention in recent years because it is the monomer of polylactic acid (PLA), which is a very promising new packaging material with biocompatibility, biodegradability, and excellent mechanical properties. PLA is expected to gradually replace polyethylene, polypropylene, polystyrene, and other non-biodegradable materials from petrochemicals, thereby reducing “white pollution” to alleviate the energy crisis [3].

*Bacillus coagulans* is a well-known probiotic used in livestock, aquaculture and human health for over five decades [4]. *B. coagulans* is also a kind of microorganism that can be used for L-LA production with the following advantages over traditional LA producing strains *Lactobacillus* and *Rhizopus oryzae*. First, the fermentation temperature of L-LA by *B. coagulans* is usually at or above 50 °C, allowing non-sterile fermentation to reduce the carbon source loss, the raw material costs, the energy consumption, and the equipment requirements [5]. Second, the L-LA produced by *B. coagulans* is usually with a high optical purity greater than 99.5% and thus expensive and can meet the needs for PLA synthesis [5]. Third, *B. coagulans* strains have a wide substrate range, and xylose can be used to produce LA [6, 7]. Given these prominent characteristics, many studies on LA production using *B. coagulans* strains have been published in the past 5 years but mainly focused on the screening of bacteria, the optimization of fermentation conditions, and the development of cheap substrates [7–11].

Product inhibition is a difficult problem in LA production [2]. With the accumulation of LA, the pH value of the fermentation broth will decrease continuously if no neutralizer is added. Calcium carbonate and sodium hydroxide are two commonly used neutralizers, and the corresponding fermentation products are calcium lactate and sodium lactate, respectively [7]. To date, the production of L-LA by using the sodium lactate process is generally lower than that by using the calcium lactate process. The inhibitory effect of sodium lactate was found stronger than that of calcium lactate [12], and lactate fermentation was found less affected by calcium lactate stress than by sodium lactate stress [13]. However, sodium lactate process is friendly to the environment and is an ideal clean LA production method because of the avoidance of gypsum (CaSO_4_) formation during L-LA recovery process [7]. Therefore, it is necessary to improve the resistance of *B. coagulans* on sodium lactate stress.

In our previous study, a high L-LA production strain, *B. coagulans* 2–6 (DSM21869), was screened, and the fermentation titer of L-LA reached 180 g/L when calcium carbonate was used as the neutralizing agent [5]. Then, the mutant strain *B. coagulans* Na-2, which has a strong sodium lactate stress resistance, was obtained through mutagenesis and screening. The titer of L-LA reached 106 g/L in 30 h by using strain Na-2, which was 10.6% higher than the 95.8 g/L obtained by using its parent strain 2–6 under sodium lactate process [14].

To investigate the molecular mechanism of the strong stress-resistance of the mutant strain *B. coagulans* Na-2 against sodium lactate, this study used genome re-sequencing and comparative genomic analysis to find genes related to lactate stress resistance and confirmed the function by genetic engineering methods.

## Results

### Identification of a mutated MFS protein by whole genome re-sequencing

After the paired-end sequencing on the Illumina HiSeq 2000 platform, 503 Mb of data was produced for *B. coagulans* Na-2. From the assembly result of the Na-2 sample, we found that the genome size was 3,075,120 bp, the guanine-cytosine (GC) content was 47.18%, the number of scaffolds was 74, and the number of contig was 4158. The assembly statistics are shown in Table S1.

**Fig. 1:**
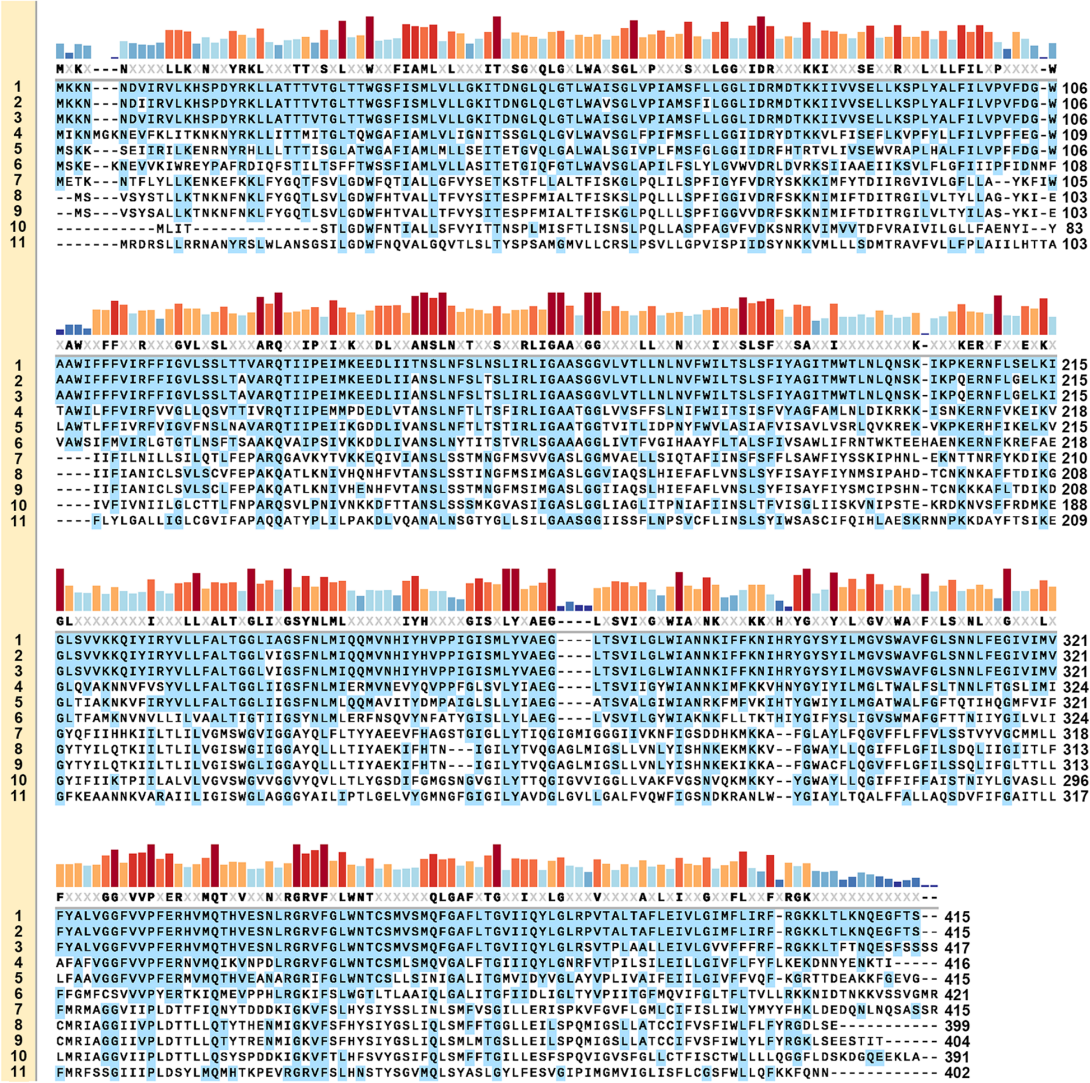
Multiple alignments of the MFS protein from *B. coagulans* 2–6 and Na-2 with the top 9 ranked proteins in BLAST search against the database of UniProtKB reference proteomes plus Swiss-Prot. Amino acids that match the reference are marked in blue. The sequence conservation is shown as colored bars. The consensus threshold is > 50%. **1** MFS-2-6 (wild MFS protein from *B. coagulans* 2–6, set as the reference sequence); **2** MFS-Na-2 (wild MFS protein from *B. coagulans* Na-2, set as the reference sequence, 97.6% identity to MFS-2-6); **3** G2TMN2 (Major facilitator superfamily MFS_1 from *B. coagulans* 36D1, 95.4% identity to MFS-2-6); **4** A0A2J6NKA8 (MFS transporter from *Nosocomiicoccus massiliensis*, 64.5% identity to MFS-2-6); **5** Q5WAS7 (Major facilitator superfamily macrolide-efflux protein from *B. clausii* KSM-K16, 57.8% identity to MFS-2-6); **6** D3G1P6 (Major facilitator superfamily macrolide-efflux protein from *Alkalihalobacillus pseudofirmus* ATCC BAA-2126, 42.6% identity to MFS-2-6); **7** A0A0H4NW12 (Macrolide-efflux protein from *B. smithii*, 22.9% identity to MFS-2-6); **8** Q81FG9 (Macrolide-efflux protein from *B. cereus* ATCC 14579, 23.8% identity to MFS-2-6); **9** A0A6L8PMK9 (Putative permease from *B. anthracis*, 24.6% identity to MFS-2-6); **10** A0A0P6VXS9 (Macrolide transporter from *Rossellomorea vietnamensis*, 24.1% identity to MFS-2-6); **11** A0A4Q4IR15 (MFS transporter from *Sporolactobacillus* sp. THM7–4, 23.7% identity to MFS-2-6)

All the 74 scaffolds were compared with the reference genome sequence of *B. coagulans* 2–6 (accession number: CP002472) by using BLAST. Finally, one gene coding for the MFS protein (accession number: AEH52936) caught our attention because multiple missense mutations were found on its gene sequence. To predict the function of the MFS protein, BLAST search against the database of UniProtKB reference proteomes plus Swiss-Prot and multiple alignment with the top 9 ranked proteins for sequence identity were performed (Fig. 1). The major facilitator superfamily macrolide-efflux protein from *B. clausii* KSM-K16 (UniProtKB number: Q5WAS7) and *Alkalihalobacillus pseudofirmus* ATCC BAA-2126 (UniProtKB number: D3G1P6) were 57.8 and 42.6% identical to the wild MFS protein (MFS-2-6). The results indicate that MFS-2-6 may be involved in the transport of macrolides.

**Fig. 2:**
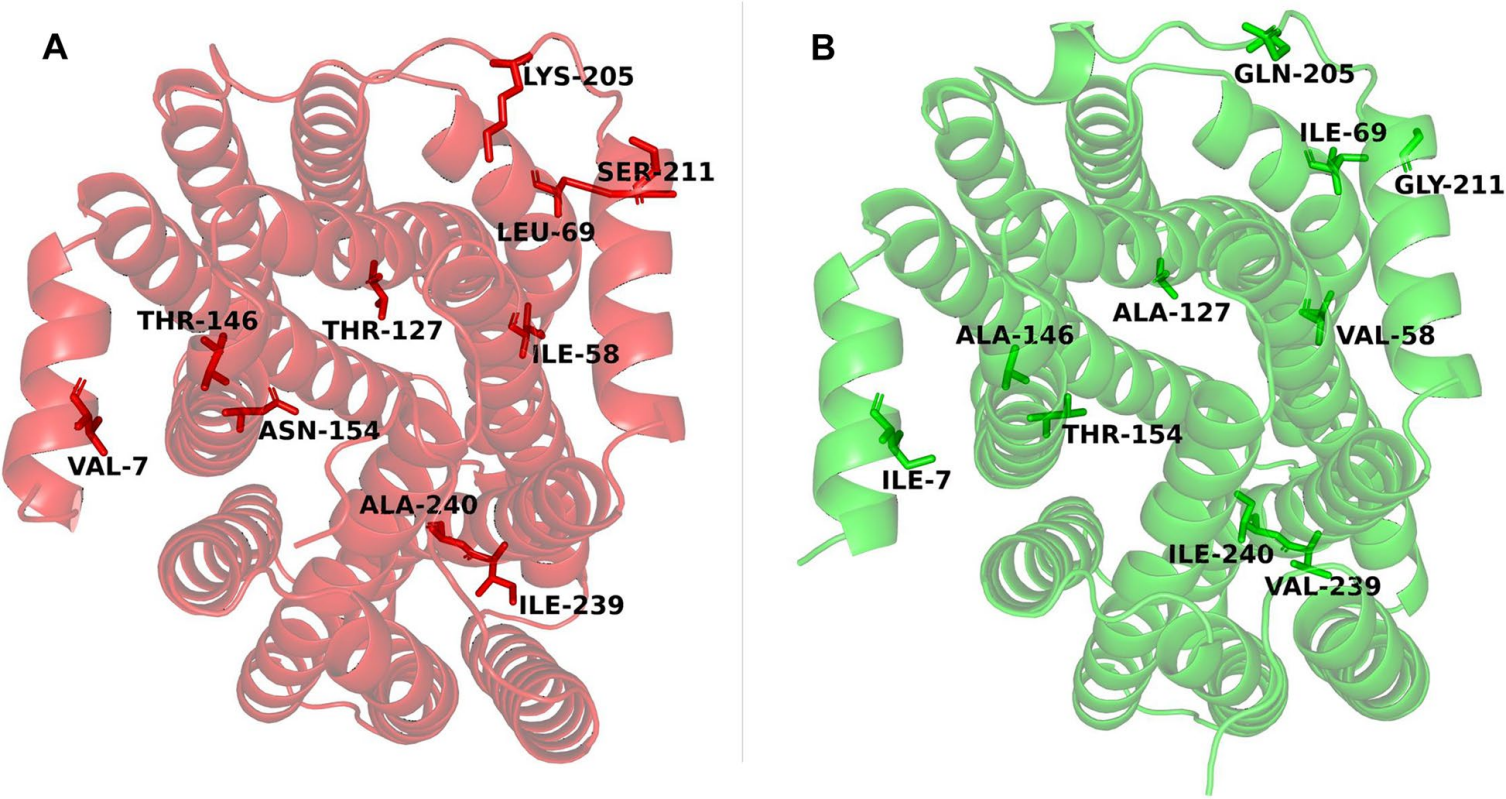
3D structure of MFS-2-6 and MFS-Na-2 predicted by tFold. **A** MFS-2-6; **B** MFS-Na-2. Amino acids at mutated sites are labeled and shown as a stick

MFS-2-6 and the mutated MFS protein (MFS-Na-2) have 415 amino acids, and 10 were different (V7I, I58V, L69I, T127A, T146A, N154T, K205Q, S211G, I239V, and A240I). The classical Swiss-Model, Phyre, the latest AlphaFold2, and Robetta were used to predict the three-dimensional (3D) structure of the MFS proteins, but the sequence consistency between the template protein used for modeling and the MFS proteins in this study is only approximately 12%. Therefore, the tFold developed by Tencent AI Lab was finally used for 3D structure prediction because this tool adopts a new set of “de novo folding” method. As shown in Fig. 2, MFS-2-6 and MFS-Na-2 contain 12 transmembrane helices, and the N- and C-terminals of the proteins are located on the intracellular side. A cavity formed in the middle of the transmembrane helices, which is the transmembrane transport channel. Mutation sites T127A and N154T are located in the center of transport channel.

### Construction and verification of the *mfs* gene heterogeneous expression strains

**Fig. 3:**
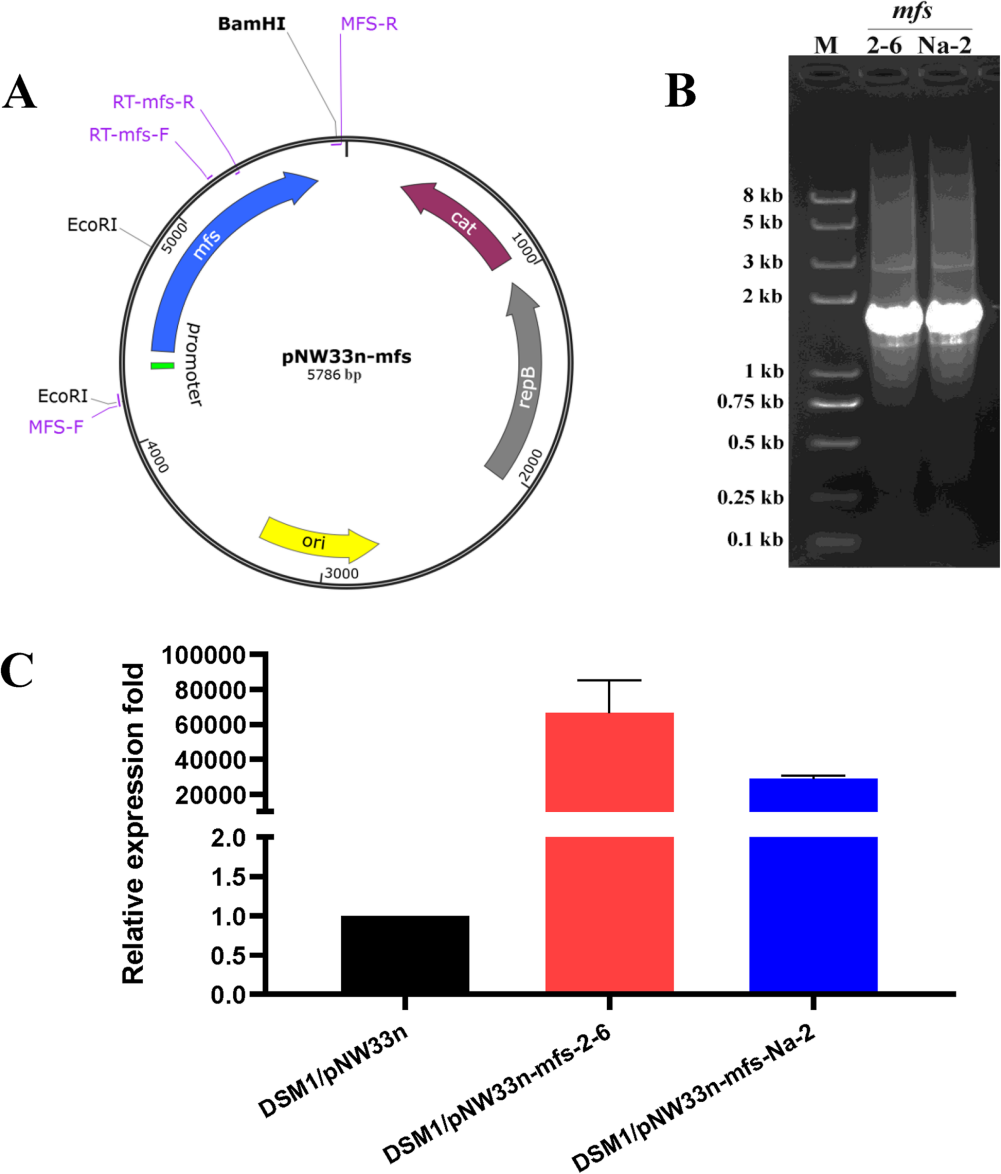
Construction of the *mfs* gene heterogeneous expression vectors and detection of *mfs* gene expression. **A** diagram of *mfs* gene heterogeneous expression vectors; **B** PCR of *mfs* gene and its native promoter; **C** detection of *mfs* gene expression by qRT-PCR

To verify the function of the MFS protein, we first wanted to knockout or overexpress the protein in wild strain *B. coagulans* 2–6. Unfortunately, all the attempts have been unsuccessful. Strain *B. coagulans* 2–6 seems genetically unmodifiable. Therefore, we were determined to verify the function of MFS in the genetically operable strain *B. coagulans* DSM1. By sequence comparison, we found no *mfs* gene on the genome of *B. coagulans* DSM1 (accession number: CP009709). Thus, overexpression of the *mfs* gene in strain DSM1 is the only way to verify its function. The diagram of the *mfs* gene heterogeneous expression vector is shown in Fig. 3A. The PCR products of the wild and mutated *mfs* genes and their native promoters are shown in Fig. 3B and Fig. S1. The expression levels of the *mfs* genes in DSM1/pNW33N-mfs-2-6 and DSM1/pNW33N-mfs-Na-2 were confirmed by qRT-PCR, and both are more than 20,000-fold higher of that in control strain *B. coagulans* DSM1/pNW33n (Fig. 3C). Therefore, the *mfs* genes were successfully expressed.

### Batch fermentation under different pH conditions

**Fig. 4:**
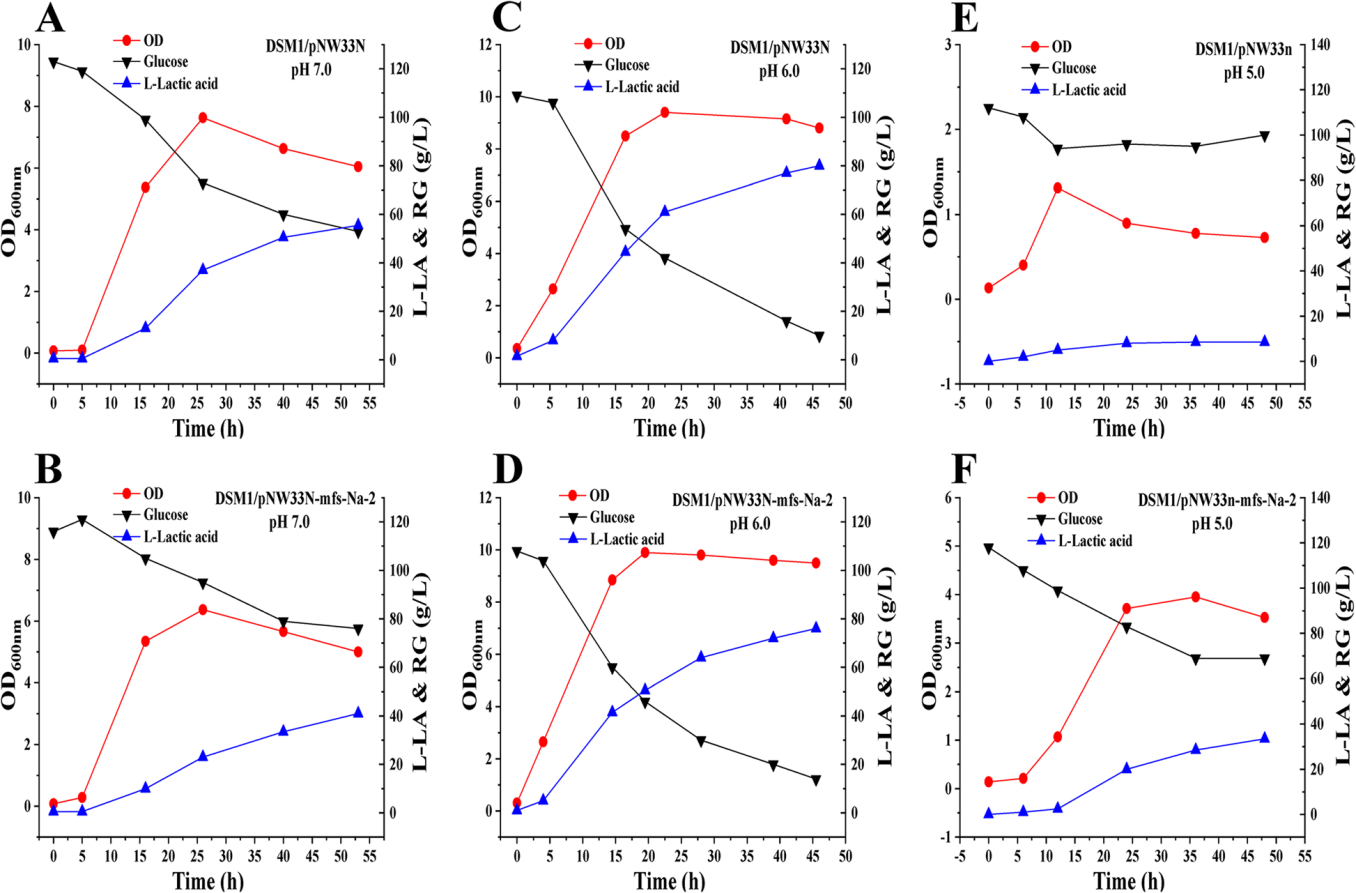
Batch fermentations of DSM1/pNW33N-mfs-Na-2 and DSM1/pNW33N under different pH conditions. **A** DSM1/pNW33N at pH 7.0; **B** DSM1/pNW33N-mfs-Na-2 at pH 7.0; **C** DSM1/pNW33N at pH 6.0; **D** DSM1/pNW33N-mfs-Na-2 at pH 6.0; **E** DSM1/pNW33N at pH 5.0; **F** DSM1/pNW33N-mfs-Na-2 at pH 5.0

To test the function of the *mfs* gene expression, the cell growth and L-LA production of the control strain *B. coagulans* DSM1/pNW33N and the mutant *mfs* gene expression strain *B. coagulans* DSM1/pNW33N-mfs-Na-2 were first compared. When the pH was controlled at 7.0, the maximum OD values of DSM1/pNW33N and DSM1/pNW33N-mfs-Na-2 were 7.63 and 6.37, respectively, and the maximum L-LA concentrations were 55.5 g/L and 41 g/L, respectively. These results indicate that the *mfs* gene expression had side effects on cell growth and L-LA production at pH 7.0 (Fig. 4A and B). When the pH was controlled at 6.0, the maximum OD values of DSM1/pNW33N and DSM1/pNW33N-mfs-Na-2 were 9.40 and 9.90, respectively, and the maximum L-LA concentrations were 80 g/L and 76 g/L, respectively. These results indicate that the *mfs* gene expression had little effect on cell growth and L-LA production at pH 6.0 (Fig. 4C and D). However, when the pH was controlled at 5.0, the maximum OD values of DSM1/pNW33N and DSM1/pNW33N-mfs-Na-2 were 1.31 and 3.95, respectively, and the maximum L-LA concentrations were 8.5 g/L and 33.5 g/L, respectively. These results indicate that the *mfs* gene expression had significant effects on cell growth and L-LA production at pH 5.0 (Fig. 4E and F).

**Fig. 5:**
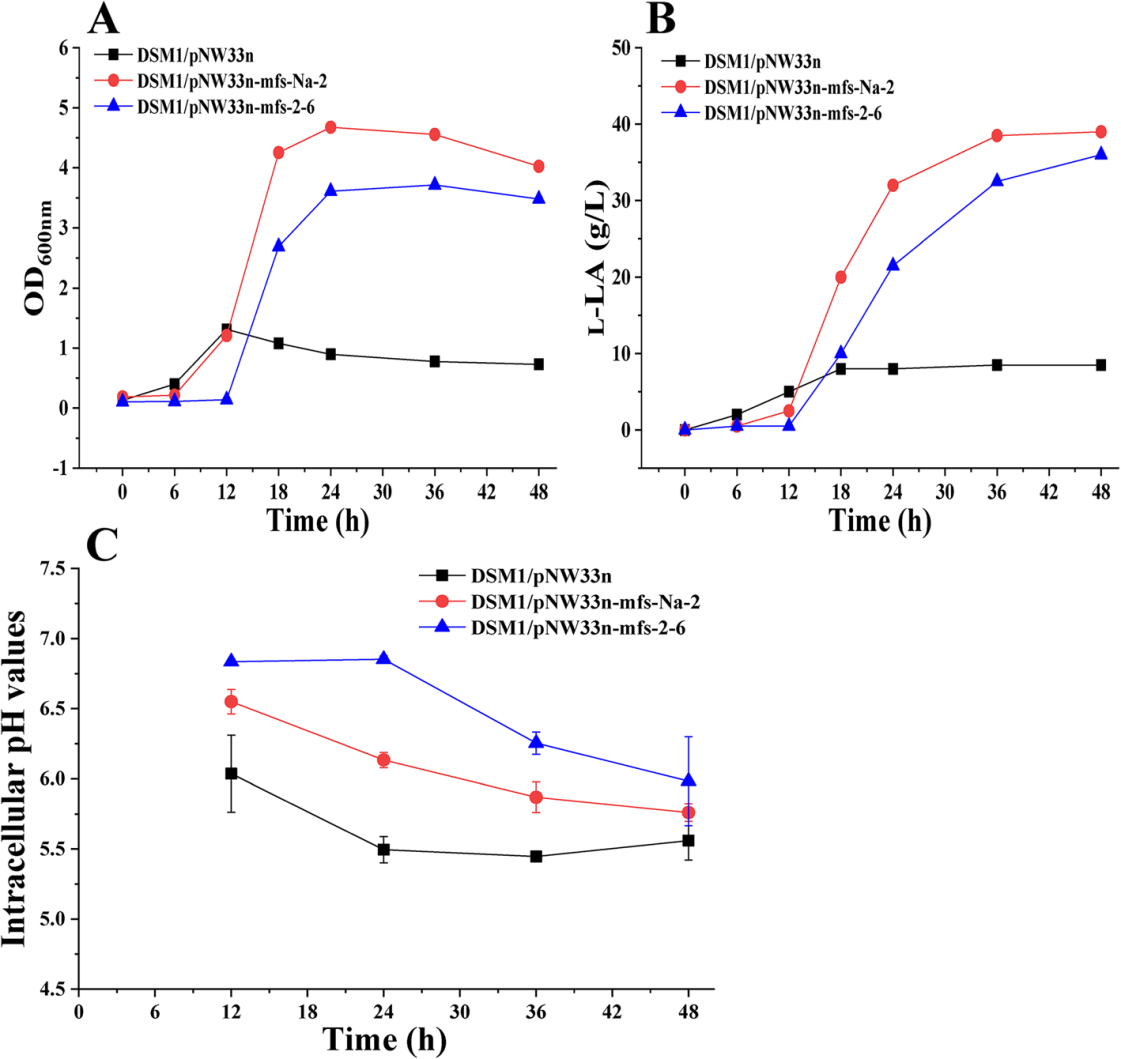
Cell growth, L-LA production, and intracellular pH comparison of DSM1/pNW33N-mfs-2-6, DSM1/pNW33N-mfs-Na-2, and DSM1/pNW33N in batch fermentations under pH 5.0. **A** cell growth comparison; **B** L-LA production comparison; **C** intracellular pH comparison

Next, the effects of mutant and wild *mfs* gene expression on cell growth and L-LA production were compared at pH 5.0. As shown in Fig. 5A, the maximum OD_600nm_ values of *B. coagulans* DSM1/pNW33N, *B. coagulans* DSM1/pNW33N-mfs-2-6, and *B. coagulans* DSM1/pNW33N-mfs-Na-2 at pH 5.0 were 1.31, 3.72, and 4.68, respectively. The maximum L-LA concentrations of *B. coagulans* DSM1/pNW33N, *B. coagulans* DSM1/pNW33N-mfs-2-6, and *B. coagulans* DSM1/pNW33N-mfs-Na-2 at pH 5.0 were 8.5, 36.0, and 39.0 g/L, respectively (Fig. 5B). These results indicate that the heterogeneous expression of the wild or the mutant *mfs* gene was beneficial to the cell growth and L-LA production of *B. coagulans* DSM1 at low pH, but the latter had better effects. The intracellular pH of the mutant and wild *mfs* gene expressing strains were approximately 0.45 and 0.69 higher than that of the control strain during the pH-controlled fermentation at 5.0 (Fig. 5C). The results suggest that the expression of the mutant *mfs* gene helped the strain maintain an intracellular pH within a suitable range, which was conducive to the L-LA production.

## Discussion

Many industrial biocatalysts suffer from low tolerance to extreme conditions. It may be beneficial to improve acid and salt tolerance for microorganisms that produce LA [15]. Therefore, obtaining genes related to LA and lactate stress and transforming strains are an important research direction. The response of strains to acid and salt stress is a complex mechanism in which various proteins play a key role, such as global transcription factor sigB [16], heat shock protein [17], and transporters [18]. Genome-wide evolution engineering strategies, such as genome shuffling [15, 19, 20], global transcription machinery engineering [21], and adaptive laboratory evolution [22–24] were used to improve acid stress resistance in microbial cells. However, to the best of our knowledge, few studies foused on *B. coagulans*. In our previously studies, comparative transcriptome and proteomic analysis of *B. coagulans* 2–6 in response to sodium lactate and calcium lactate were performed and many genes were found related to lactate stress [11, 12]. However, no single overexpression of any gene can improve the LA and lactate resistance of the strain. *B. coagulans* Na-2, which is highly resistant to lactate stress, was obtained through mutagenesis screening in our laboratory [14]. In this study, a mutated MFS protein (MFS-Na-2) was found by genome sequencing and comparative genomics methods and its function in LA production was verified.

The MFS is one of the world’s largest families of secondary active membrane transporters. As a result of the diversity of its substrates, the MFS plays an important role in the exchange of solutes and the metabolism of energy [25]. The function of MFS-Na-2 in L-LA production by *B. coagulans* was confirmed by the heterologous expression (Fig. 5), but only in an acidic environment (pH 5.0). The high intracellular pH of the MFS expressing strains indicates that H^+^ was involved in substrate transport, like many other MFS superfamily proteins, such as lactose/H^+^ symporter LacY [26], fucose/H^+^ symporter FucP [27], and multidrug/H^+^ antiporter [28]. The expression of MFS-Na-2 made the intracellular pH of the strain higher than that of the control strain but lower than that of wild-type MFS-2-6, suggesting that an appropriate intracellular pH range is beneficial to cell growth and L-LA production.

Unfortunately, the transport substrate of MFS-2-6 and MFS-Na-2 was difficult to identify. MFS transporters often bind their substrates weakly (high μM to mM range) to transport them across membranes at physiologically relevant concentrations [25]. Therefore, the virtual docking based on the 3D structure is also inaccurate. The results of BLAST analysis show that the protein is very likely a macrolide transporter (Fig. 1). Among the 10 mutation sites between MFS-2-6 and MFS-Na-2, T127A and N154T are in the center of the transmembrane transport channel, which will inevitably affect the binding of the proteins to the substrate.

## Conclusions

The MFS protein identified in this paper can improve the ability of *B. coagulans* to resist acidic environments, keep the intracellular pH within a suitable range, and produce more L-LA. The effect may be achieved by transporting H^+^ from the intracellular side to the extracellular side while transporting its substrate. The MFS protein has an application potential in environment-friendly L-LA production.

## Materials and methods

### Strains, plasmids, and primers

The bacterial strains, plasmids, and primers used in this study are listed in Table 1. *B. coagulans* strains were grown in Luria–Bertani (LB) medium at 50 °C unless otherwise stated. *Escherichia coli* strains were aerobically grown in LB medium at 37 °C on a rotary shaker. The antibiotics used for *E. coli* were 100 μg/mL ampicillin and 34 μg/mL chloramphenicol, while 7 μg/mL chloramphenicol was used for *B. coagulans*.

**Table 1:** Strains, plasmids, and primers used in this study

Strain, plasmid or primer	Characteristics, or sequence	Source or reference
**Strains**		
*E. coli* JM109	*recA1*, *endA1*, *gyrA96*, *thi-1*, *hsdR17 (rk*^*−*^*mk*^*+*^*)*, *e14*^*−*^*(mcrA*^*−*^*) supE44*, *relA1*, Δ (*lac*-*proAB*)/F′[*traD36*, *proAB*^+^, *lacI*^*q*^, *lacZ*ΔM15]	Takara
*B. coagulans* 2–6	Wild strain isolated from soil, DSM21869	5
*B. coagulans* Na-2	Mutant strain of *B. coagulans* 2–6 with high sodium lactate tolerance ability	14
*B. coagulans* DSM1	Type strain, transformation host	DSMZ
*B. coagulans* DSM1/pNW33n	Control strain, *B. coagulans* DSM1 harboring pNW33n	This study
*B. coagulans* DSM1/pNW33n-mfs-2-6	Heterogeneous expression strain, *B. coagulans* DSM1 harboring pNW33n-mfs-2-6	This study
*B. coagulans* DSM1/pNW33n-mfs-Na-2	Heterogeneous expression strain, *B. coagulans* DSM1 harboring pNW33n-mfs-Na-2	This study
**Plasmids**		
pNW33n	4.2 kb, Cm^r^, *Geobacillus*-*E. coli* shuttle vector	29
pNW33n-mfs-2-6	Cm^r^, pNW33n carrying native *mfs* gene from *B. coagulans* 2–6 and its promotor	This study
pNW33n-mfs-Na-2	Cm^r^, pNW33n carrying mutated *mfs* gene from *B. coagulans* Na-2 and its promotor	This study
Primers^*a*^		
MFS-F	5′-TCTGATTGTGAAATTGAATTCAGCAAGCCCTAAGTATGTAAAATAC-3’	This study
MFS-R	5′-CTGCGGTGCGGCCATGGATCCTAACGCACCTTTGTTGTCTGCT-3’	This study
RT-mfs-F	5′-ACGTTGCCGAAGGTTTGACT-3’	This study
RT-mfs-R	5′-AACACAGCCCAGCTTACTCC-3’	This study
RT-16S-F	5′-GGGAAACCGGGGCTAATACC-3’	This study
RT-16S-R	5′-TCTGTAAGTGGCAGCCGAAG-3’	This study

^*a*^The underlined letters indicate the introduction of restriction sites

### DNA manipulations

Genomic DNA was extracted using the Wizard Genomic DNA Purification Kit (Promega, USA). Plasmid DNA was isolated using the *Easypure* Plasmid Miniprep Kit (Transgen, China). The QIAquick Gel Extraction Kit (Qiagen, Germany) was used for DNA purification. Polymerase chain reaction (PCR) was performed by using PrimeSTAR® HS DNA Polymerase (TaKaRa, China), and the primers were prepared by Sangon Biotech (Shanghai, China). The pEASY®-Basic Seamless Cloning and Assembly Kit (Transgen, China) was used to integrate the vector and the DNA inserts. The total RNA was extracted with an EasyPure RNA Purification Kit (Transgen, China). The qRT-PCR reactions were performed using the TransScript II Green One-Step qRT-PCR SuperMix (Transgen, China) on a LightCycler 96 instrument (Roche, Germany).

### Whole genome re-sequencing of *B. coagulans* Na-2

The genomic DNA of *B. coagulans* Na-2 was extracted and used for DNA sequencing. Sequencing was carried out on the Illumina HiSeq 2000 platform (Beijing Genomics Institute, Beijing, China). The filtered short reads were assembled into a genomic sequence using SOAPdenovo [29]. Single Nucleotide Polymorphisms (SNPs) were detected based on the aligned result of the assembly sequence and the reference genome sequence of *B. coagulans* 2–6 (accession number: CP002472) by using BLAST. The sequencing data of *B. coagulans* Na-2 have been deposited in the NCBI Sequence Read Archive under accession number SRR9887488.

### Transformation of *B. coagulans*

A modified protocol for electroporation of *B. coagulans* DSM1 (for pNW33n and its derivatives) has been applied [30]. The DSM1 strain was grown overnight at 45 °C in 20 mL of LB medium in a 100-mL flask without shaking. The overnight culture was inoculated (10% vol/vol) into 50 mL of LB medium in a 500-mL flask. The cells were incubated at 45 °C with shaking at 150 rpm until the optical density (OD) at 600 nm reached approximately 0.9–1.0. The cells were collected by centrifugation at 4 °C, washed three times with 40, 20, and 10 mL of ice-cold GS medium (0.4 mol/L of sorbitol, 4 mmol/L of MgCl_2_, 5 mmol/L of KH_2_PO_4_, 10% glycerol), resuspended in 100 μL of ice-cold GS medium, and used immediately for electroporation. An ice-cold electroporation cuvette (1-mm gap) was filled with 75 μL of cell suspension mixed with 0.2 μg of plasmid DNA and kept on ice for 2 min. The electroporation condition was set as a square wave at 1.6 KV (BioRad electroporator; BioRad Laboratories, Hercules, CA). After electroporation, the cells were transferred into 1 mL of prewarmed (50 °C) RG medium (LB medium with 0.5 M of sucrose, 55.6 mM of glucose, and 20 mM of MgCl_2_). These cells were incubated for 3 h at 45 °C and plated on medium containing chloramphenicol.

### Construction of the *mfs* gene heterogeneous expression strain

The *mfs* gene and the promoter (Fig. S2) of *B. coagulans* 2–6 and Na-2 were amplified by PCR using primers MFS-F and MFS-R (Table 1). The 1.5 kb purified DNA fragment was integrated between the *Eco*RI and *Bam*HI restriction sites of *E. coli*/*B. coagulans* shuttle vector pNW33n [31] and transformed into *E. coli* JM109. The recombinant plasmids were isolated and verified by DNA sequencing. Then, the recombinant plasmids were transformed into *B. coagulans* DSM1 using the above-mentioned protocol, resulting in *mfs* gene heterogeneous expression strains *B. coagulans* DSM1/pNW33n-mfs-2-6 and *B. coagulans* DSM1/pNW33n-mfs-Na-2. The control strain, *B. coagulans* DSM1/pNW33n, was also constructed by introducing pNW33n into *B. coagulans* DSM1.

### Detection of *mfs* gene expression by quantitative real-time PCR (qRT-PCR)

qRT-PCR was used to confirm the *mfs* gene expression in *B. coagulans* transformants. The RT-mfs-F and RT-mfs-F primers were used for the *mfs* gene, and RT-16S-F and RT-16S-R for the reference gene, as shown in Table 1. The relative gene expression folds were analyzed using the 2^-ΔΔCt^ method [32]. qRT-PCR runs were conducted with three biological and three technical replicates on each sample.

### Protein analysis

The BLAST search was performed against the database of UniProtKB reference proteomes plus Swiss-Prot (https://www.uniprot.org/blast/). Sequence analysis was performed with the SnapGene software (Version 5.3.3, GSL Bio-tech, America). MUSCLE (Hinxton, UK) was used to create sequence alignments. The 3D structure of the identified MFS proteins was predicted by tFold (https://drug.ai.tencent.com/console/cn/tfold?type=predict), which was developed by Tencent AI Lab, and visualized by using the PyMol software (Version 2.5.0a0, Schrödinger, LLC, America).

### Batch fermentation

Single colonies of *B. coagulans* strains were picked up and cultivated in GY medium (40 g/L of glucose, 10 g/L of yeast extract, 1 g/L of CaCl_2_, pH 6.7) with chloramphenicol for 24 h at 50 °C, with shaking at 150 rpm on a rotary shaker. Then, 2.5 mL of bacterial liquid was inoculated into 50 mL of fresh GY medium and incubated at 50 °C until the OD value at 600 nm reached 1. The seed culture was then inoculated into a bioreactor (inoculum volume at 5%) for L-LA production. All batch fermentations were conducted at 50 °C in a 2-L bioreactor (BioFlo/CelliGen 115, Eppendorf, Germany) containing 1 L of fermentation medium (130 g/L of glucose, 10 g/L of yeast extract, 1 g/L of CaCl_2_). The agitation was controlled at 200 rpm, and no aeration was supplied. The pH was controlled at 5.0, 6.0, and 7.0 by adding 10 M of sodium hydroxide. Samples were taken every few hours, and the cell growth was monitored by testing OD_600nm_. An SBA-40C biosensor analyzer (Institute of Biology, Shandong Academy of Sciences, China) was used to measure residual glucose (RG) and L-LA concentrations.

### Intracellular pH (pH_in_) determination

To explore the function of MFS, the pH_in_ values of *B. coagulans* DSM1 derivative strains were determined by using 2′,7′-bis-(2-carboxyethyl)-5(and 6)-carboxyfluorescein succinimidyl ester (BCECF-AM) as the fluorescence probe [33]. Cells were taken during pH-controlled fermentation, washed with normal saline, and diluted to an OD_600_ of 0.8. After centrifugation, the collected cells were suspended in 50 mM of potassium phosphate buffer solution (pH 7.0) and added with BCECF-AM to a final concentration of 5 μM. The cells were dyed in a 28 °C incubator for 3 h in the dark.

The stained cells were suspended and diluted in 100 mM of potassium phosphate buffer solution (pH 4.0, 4.5, 5.0, 5.5, 6.0), resulting in a thallus OD_600_ of approximately 0.4. To make the intracellular pH consistent with the extracellular pH, a certain amount of H^+^/K^+^ ion carrier Nigeritin was added to the final concentration of 30 μM, and the solution was placed in a dark chamber for 60 min. The treated bacterial suspension was divided into two parts. The fluorescence intensity at the excitation wavelength of 490 and 440 nm was measured by a multi-function microplate analyzer with the emission wavelength of 530 nm. The fluorescence intensity was calculated as the total fluorescence intensity at 490 and 440 nm and respectively named as tF490 and tF440. To measure the intracellular fluorescence intensity, the extracellular fluorescence interference should be removed. Therefore, the other part of the bacterial suspension was filtered by a 0.22-μm filter membrane, and 200 μL of the filtrate was added to the black plate. The fluorescence intensity of the filtrate at the 490 and 440 nm excitation wavelengths was measured as bF490 and bF440, respectively. The relative fluorescence intensity (RFI) was calculated as follows: RFI = (tF490-bF490) / (tF440-bF440). The standard curve of RFI and intracellular pH was established using pH as the ordinate and the fluorescence intensity ratio as the abscissa.

## Supplementary Information

**Additional file 1: Table S1.** Assembling statistics of *B. coagulans* Na-2. **Figure S1.** The original and unprocessed version of Fig. 3B. **Figure S2.** The description and assembled sequences used in this study.

## Data Availability

The sequencing data generated and analyzed in this study are available at NCBI (https://www.ncbi.nlm.nih.gov) under accession numbers SRR9887488.
