# Supplementary material for: Identification of a major facilitator superfamily protein that is beneficial to L-lactic acid production by Bacillus coagulans at low pH
Source: BMC Microbiol. 2022 Dec 20;22:310. doi: 10.1186/s12866-022-02736-2 (PMC9764580; doi:10.1186/s12866-022-02736-2)
Supplement: Supplementary file 1 — Additional file 1: Table S1. Assembling statistics of B. coagulans Na-2. Figure S1. The original and unprocessed version of Fig. 3B. Figure S2. The description and assembled sequences used in this study. [file 12866_2022_2736_MOESM1_ESM.docx]

**Supplementary Material**

**Identification of a major facilitator superfamily protein that is beneficial to L-lactic acid production by *Bacillus coagulans* at low pH**

Wenzhe Tian, Jiayang Qin^*^, Congcong Lian, Qingshou Yao, Xiuwen Wang

College of Pharmacy, Binzhou Medical University, Yantai 264003, People's Republic of China

*Correspondence: qinjy@bzmc.edu.cn

**Table S1.** Assembling statistics of *B. coagulans* Na-2.

|  | **Scaffold** | **Contig** |
| --- | --- | --- |
| **Total Number (#)** | 74 | 4,158 |
| **Total Length (bp)** | 3,075,120 | 2,416,794 |
| **N50 (bp)** | 103,992 | 703 |
| **N90 (bp)** | 29,268 | 296 |
| **Max Length (bp)** | 370,977 | 5,996 |
| **Min Length (bp)** | 504 | 200 |
| **GC Content (%)** | 47.18 | 47.18 |

**Figure S1** The original and unprocessed version of Fig. 3B.

**
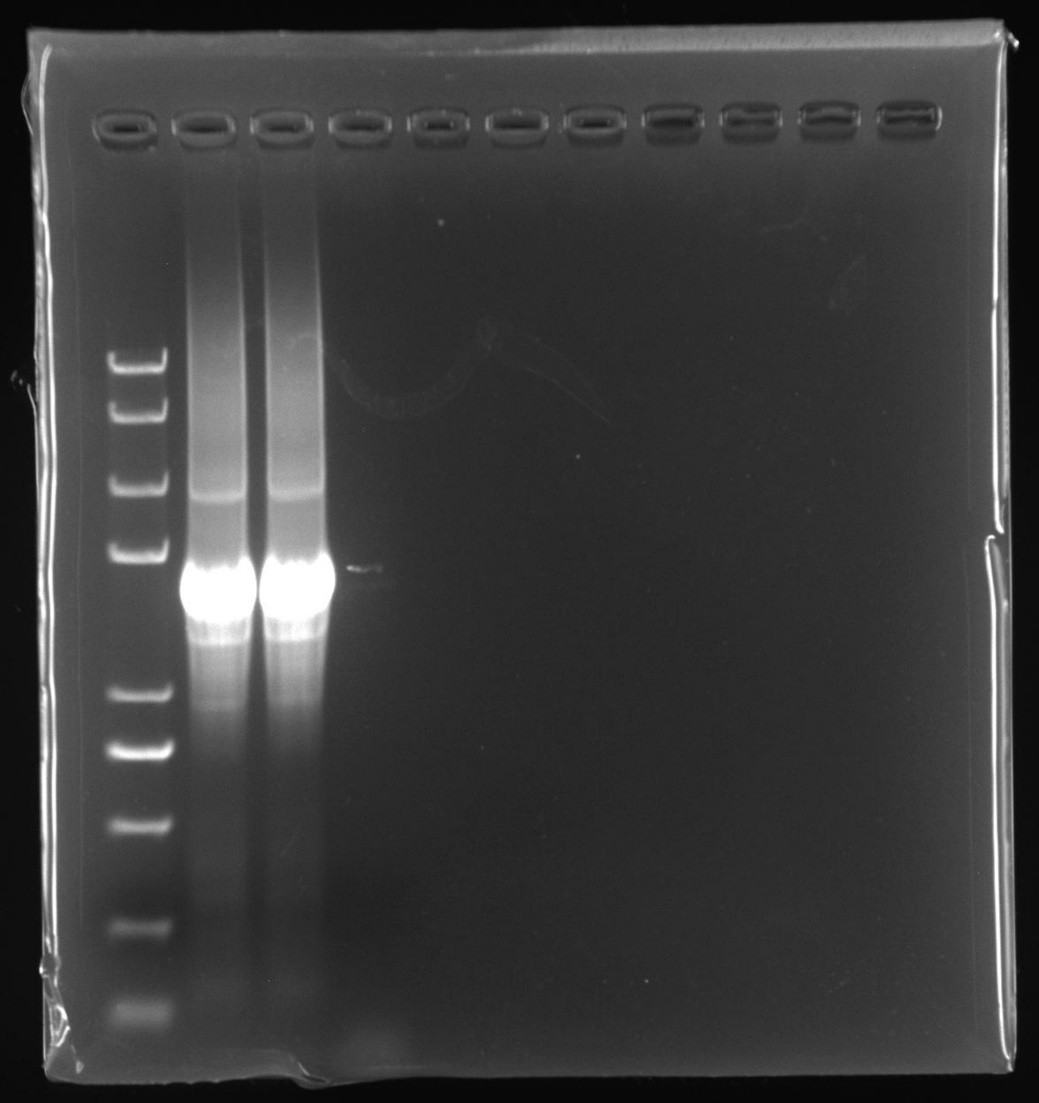
**

**Figure S2** The description and assembled sequences used in this study.

**Sequence of *mfs-2-6***

(5′ flank-EcoRI-native promoter-*mfs-2-6* gene-BamHI-3′ flank)

tctgattgtgaaattgaattcagcaagccctaagtatgtaaaataccccggaaacgggcccggcccaaaatagcaggcaggacacttgacgttttgaaaatataaaacatttccccgaaacccgctgaaaacggctgttctaatattgaaaaattttccccagccttgtaaaatggaaaaaaggagttgcaattgaatgaaatataaaaatagttcggacttgatcatgcgatgaaaaaaaacaatgatgttatacgagtgttaaagcattcgcccgactatagaaagttattggcaacgactaccgttacagggttaacaacctggggttcctttatttcaatgctcgtccttttagggaaaataacggataacggattgcagttagggacgctgtgggctataagcggtttagtgccgatcgcgatgagttttctattaggcgggcttattgacagaatggataccaagaaaatcattgtagtatctgaactgctgaagtcgccgttatatgccctatttatacttgtgcccgtgttcgacggttgggcagcttggatttttttctttgtgatcaggttttttatcggcgttctctcatctttgactaccgttgcgagacaaaccattattccggaaatcatgaaagaagaagacttaatcatcacgaattcattaaacttttctttgaacagtttgattcgcttaatcggtgcagcatccggtggggttttggtaacattgctcaatctaaatgtcttctggatcttaacatctttatcttttatttacgccggcatcaccatgtggacattgaatttacaaaattccaaaataaagccaaaggaaagaaactttctcagcgaactaaagattggcttatccgtggtaaaaaagcaaatttacatcagatatgtattgctttttgccttaacagggggcttaattgccgggtcttttaacctgatgattcaacaaatggtaaaccatatttatcatgttccccctattggcattagcatgttatacgttgccgaaggtttgacttctgtcattctgggtctttggattgccaataataaaatttttttcaaaaatattcaccggtatggctatagctatatcttaatgggagtaagctgggctgtgtttgggctttccaataatttgtttgaaggcattgtcattatggtcttctatgcacttgtcggtggattcgttgtcccgttcgaacggcatgtgatgcaaacacatgttgaaagcaatcttagagggagagttttcgggctttggaatacttgcagcatggtctccatgcaatttggtgcttttctgacaggggttatcattcaatatcttggacttaggccagtaacggcacttactgctttcctggagatcgtactcgggataatgtttttaattcgattcagaggaaagaaattaacattaaaaaaccaagaaggctttacttcttgattttttaatctatataaaatgaacttaagtttttgcaacaaggtcgagcaccaatttgcatgatgactacgttttacagcagacaacaaaggtgcgttaggatccatggccgcaccgcag

**Sequence of *mfs-Na-2***

(5′ flank-EcoRI-native promoter-*mfs-Na-2* gene-BamHI-3′ flank)

tctgattgtgaaattgaattcagcaagccctaagtatgtaaaataccccggaaacgggcccggcccaaaatagcaggcaggacacttgacgttttgaaaatataaaacatttccccgaaacccgctgaaaacggctgttctaatattgaaaaattttccccagccttgtaaaatggaaaaaaggagttgcaattgaatgaaatataaaaatagttcggacttgatcatgcgatgaaaaaaaacaatgatgttatacgagtgttaaagcattcccccgactatagaaagttattggcaacgactaccgttacaggattaacaacctggggttcttttatttcaatgctcgtccttttagggaaaataacggataacggattgcagttagggacgctgtgggctgtaagcggtttagttccgattgcgatgagttttatattaggcgggctcatcgacagaatggataccaagaaaatcattgtagtatctgaactgctgaaatctccgctatatgccctttttatacttgtgcccgtgttcgacggttgggcagcttggatttttttctttgtgatcaggttttttatcggcgttctctcatctttgactgccgttgcgagacaaaccattattccggaaatcatgaaagaagaagacttgatcatcgcgaattcattgaatttttctttgaccagtttgattcgcttaatcggtgcagcttccggcggggttttggtaacattgctcaatctaaatgtcttctggatcttaacatctttatcttttatttacgccggcatcaccatgtggacattgaatttacaaaattccaaaataaagccacaggaaagaaactttctcggtgaactaaaaattggcttatccgtagtaaaaaaacaaatttacatcagatatgtattgctttttgccttaacaggtggcttagttatcgggtcttttaacctgatgattcaacaaatggtaaaccatatttatcatgttccccctattggcattagcatgttatacgttgccgaaggtttgacttctgtcattctgggtctttggattgccaataataaaatttttttcaaaaatattcaccggtatggctatagctatatcttaatgggagtaagctgggctgtgtttgggctttccaataatttgtttgaaggcattgtcattatggtcttctatgcacttgtcggtggattcgttgtcccgttcgaacggcatgtgatgcaaacacatgttgaaagcaatcttagagggagagttttcgggctttggaatacttgcagcatggtctccatgcaatttggtgcttttctgacaggggttatcattcaatatcttggacttaggccagtaacggcacttactgctttcctggagatcgtactcgggataatgtttttaattcgattcagaggaaagaaattaacattaaaaaaccaagaaggctttacttcttgattttttaatctatataaaatgaacttaagtttttgcaacaaggtcgagcaccaatttgcatgatgactacgttttacagcagacaacaaaggtgcgttaggatccatggccgcaccgcag
